# Characteristics and outcomes of patients with COVID‐19 admitted to ICU in a tertiary hospital in Stockholm, Sweden

**DOI:** 10.1111/aas.13694

**Published:** 2020-09-15

**Authors:** Emma Larsson, Olof Brattström, Christina Agvald‐Öhman, Jonathan Grip, Francesca Campoccia Jalde, Kristoffer Strålin, Pontus Nauclér, Anders Oldner, David Konrad, Björn P. Persson, Lars I. Eriksson, Johan Mårtensson, Karolinska Intensive Care COVID‐19 Study Group

**Affiliations:** ^1^ Department of Physiology and Pharmacology Karolinska Institutet Stockholm Sweden; ^2^ Department of Perioperative Medicine and Intensive Care Karolinska University Hospital Stockholm Sweden; ^3^ Department of Clinical Science Intervention and Technology (CLINTEC) Karolinska Institutet Stockholm Sweden; ^4^ Department of Infectious Diseases Karolinska University Hospital Stockholm Sweden; ^5^ Department of Medicine, Huddinge Karolinska Institutet Stockholm Sweden; ^6^ Division of Infectious Diseases Department of Medicine, Solna Karolinska Institutet Stockholm Sweden

**Keywords:** ARDS, COVID‐19, critical care, SARS‐CoV‐2

## Abstract

**Background:**

Information on characteristics and outcomes of intensive care unit (ICU) patients with COVID‐19 remains limited. We examined characteristics, clinical course and early outcomes of patients with COVID‐19 admitted to ICU.

**Methods:**

We included all 260 patients with COVID‐19 admitted to nine ICUs at the Karolinska University Hospital (Stockholm, Sweden) between 9 March and 20 April 2020. Primary outcome was in‐hospital mortality among patients with definite outcomes (discharged from ICU or death), as of 30 April 2020 (study end point). Secondary outcomes included ICU length of stay, the proportion of patients receiving mechanical ventilation and renal replacement therapy, and hospital discharge destination.

**Results:**

Of 260 ICU patients with COVID‐19, 208 (80.0%) were men, the median age was 59 (IQR 51‐65) years, 154 (59.2%) had at least one comorbidity, and the median duration of symptoms preceding ICU admission was 11 (IQR 8‐14) days. Sixty‐two (23.8%) patients remained in ICU at study end point. Among the 198 patients with definite outcomes, ICU length of stay was 12 (IQR, 6‐18) days, 163 (82.3%) received mechanical ventilation, 28 (14.1%) received renal replacement therapy, 60 (30.3%) died, 62 (31.3%) were discharged home, 47 (23.7%) were discharged to ward, and 29 (14.6%) were discharged to another health care facility. On multivariable logistic regression analysis, older age and admission from the emergency department was associated with higher mortality.

**Conclusion:**

This study presents detailed data on clinical characteristics and early outcomes of consecutive patients with COVID‐19 admitted to ICU in a large tertiary hospital in Sweden.

**Editorial Comment**

In this important, timely, and well‐conducted study, clinical characteristics and outcomes of 260 patients with COVID‐19 admitted to the intensive care units at the Karolinska University Hospital, Stockholm, are presented.

## 1 INTRODUCTION

The first patient with confirmed coronavirus disease 2019 (COVID‐19), caused by the severe acute respiratory syndrome coronavirus 2 (SARS‐CoV‐2), admitted to a Swedish intensive care unit (ICU) was reported on 6 March 2020. As of 20 April 2020, a total of 1100 patients with COVID‐19 had been treated in Swedish ICUs, according to the Swedish Intensive Care Registry.^1^ Approximately one quarter of these patients were admitted to the Karolinska University Hospital ICUs.

Data on baseline characteristics and outcomes of ICU patients with COVID‐19 are essential for planning actions preceding local outbreaks and to assess the need of rehabilitation in ICU survivors. Recent reports from China, Italy and the US indicate a mortality rate between 60% and 85% among ICU patients with data on definite outcomes (discharged alive from ICU or death).^2^, ^3^, ^4^ Differences in patient characteristics and socioeconomic status, health care systems, ICU admission thresholds and availability of ICU beds between countries might explain such wide differences in mortality.^5^, ^6^, ^7^ The aim of the current study was to examine clinical characteristics and outcomes of ICU‐treated patients with COVID‐19 in a large tertiary hospital in Stockholm, Sweden.

## 2 METHODS

This study was approved by the Swedish Ethical Review Authority (approval number 2020‐01477) with a waiver of informed consent. The study has been performed in accordance with the ethical standards laid down in the 1964 Declaration of Helsinki and its later amendments.

### 2.1 Study population

The study was conducted at the Karolinska University Hospital, the largest (1600 beds) academic hospital in Stockholm, Sweden. As for other ICUs in Sweden, all intensive care beds are funded by the government. During the COVID‐19 pandemic, the Karolinska University Hospital expanded intensive care from three to nine ICUs (from 38 to 182 ICU beds). We included all consecutive patients admitted to any of the ICUs at the Karolinska University Hospital in Stockholm, Sweden with confirmed severe acute respiratory syndrome coronavirus 2 (SARS‐CoV‐2) by polymerase chain reaction, between 9 March 2020 and 20 April 2020.

### 2.2 Data collection

We included routine clinical data prospectively recorded and validated in our local ICU COVID‐19 quality registry. Registry data are retrieved from the Swedish Intensive Care Registry and from available electronic patient data management systems (Clinisoft [GE, Barringgton IL] and the Karolinska internal data warehouse, including data from Take Care [CompuGroup Medical, Koblenz, Germany]). In this study, we included all consecutive ICU patients with COVID‐19 recorded in the local ICU COVID‐19 quality registry from 9 March until 20 April 2020. Study end point was 30 April 2020.

Collected data included patient demographics, comorbidities, body mass index (BMI), duration of symptoms preceding ICU admission, medication associated with coronavirus disease administered before and/or during ICU admission, mechanical ventilation, renal replacement therapy, ICU length of stay, discharge destination and mortality. Information on comorbidities was obtained (from previous medical records, chronic medications, and/or from patients or relatives) and recorded by the treating clinician. There was no missing baseline admission data except for BMI (missing data for 30 patients). Clinical outcomes were available for all patients.

### 2.3 Outcomes

The primary outcome was in‐hospital mortality, at study end point (30 April 2020). Secondary outcomes included ICU length of stay, the proportion of patients receiving mechanical ventilation and renal replacement therapy, and hospital discharge destination. During the review process, an extended follow‐up time of mortality was requested for all patients. Therefore, we included 120‐day mortality as a secondary outcome measure.

### 2.4 Statistics

Categorical variables are presented as number (with percentages). Mortality is also presented as proportions (with 95% confidence intervals) in subgroups. Continuous variables are summarized as median with interquartile range (IQR). Data was analyzed using STATA version 15.1 (Stata Corp., College Station, TX). Outcome data were presented in categories for age (<40 years; 40‐49 years; 50‐59 years; 60‐69 years; and ≥ 70 years), comorbidity (no comorbidity; 1 comorbidity; 2 comorbidities; >2 comorbidities; hypertension; cardiac disease; chronic obstructive pulmonary disease (COPD) or asthma; immune deficiency; liver disease; kidney disease; diabetes (type 1 or 2); and neuromuscular disease), BMI (<25 kg/m^2^; 25‐29 kg/m^2^; 30‐34 kg/m^2^; and ≥ 35 kg/m^2^), symptom duration before ICU admission (<5 days; 5‐9 days; 10‐14 days; 15‐19 days; and ≥ 20 days), location before ICU admission (other ICU, emergency department and in‐hospital admission) and chloroquine phosphate use (yes or no). Time to death until 30 April 2020 was presented using the Kaplan‐Meier methodology. Assessment of the following potential risk factors for in‐hospital mortality was performed using multivariable logistic regression analysis: age, sex, symptom duration (log‐transformed to achieve normality distribution), number of comorbidities (none vs. ≥1), location before ICU admission (ward or other ICU vs emergency department), and chloroquine phosphate use. Model discrimination was determined by the area under the curve. Additionally, time to death within 120 days was assessed using multivariable Cox regression analysis and presented using the Kaplan‐Meier methodology. Proportional hazard assumption was assessed using the Shoenfeld Residuals test. A two‐sided *P* Value < 0.05 was considered statistically significant.

## 3 RESULTS

**Figure 1:**
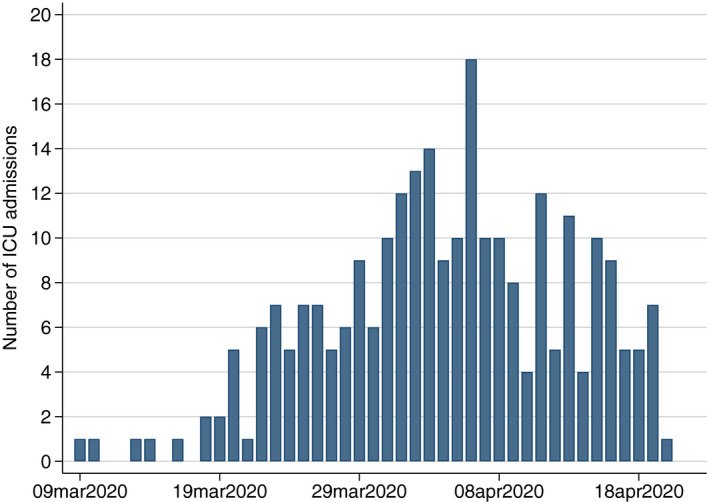
Daily number of new ICU admissions with COVID‐19

A total of 260 patients (80% male; median age 59 [IQR 51‐65] years), including three pregnant women, with severe COVID‐19 were admitted to our ICUs between 9 March 2020 and 20 April 2020 (Figure 1). The majority (68.5%) were in‐hospital admissions and 20.4% were admitted from other ICUs to optimize regional ICU resource utilization. The median BMI was 28 (IQR 26‐32) kg/m^2^. The median duration of symptoms before ICU admission was 11 (IQR 8‐14) days. Overall, 106 (40.8%) patients had no known comorbidity. The most prevalent comorbidities included hypertension (39.6%), diabetes (type 1 or 2; 26.2%) and COPD or asthma (12.7%). Approximately one‐third had only one comorbidity and one‐quarter had two or more comorbidities (Table 1).

**Table 1:** Baseline characteristics

Characteristic	All patients (n = 260)
Age, years	59 (51‐65)
Male sex	208 (80.0%)
Body mass index, kg/m^a^	28 (26‐32)
Location before ICU admission	
In hospital admission	178 (68.5%)
Other ICU	53 (20.4%)
Emergency department	29 (11.2%)
Time from symptom to ICU admission, days	11 (8‐14)
Pregnancy	3 (1.2%)
Comorbidities	
No comorbidity	106 (40.8%)
One comorbidity	80 (30.8%)
Two comorbidities	59 (22.7%)
More than two comorbidities	15 (5.8%)
Chronic hypertension	103 (39.6%)
Chronic cardiac disease	17 (6.5%)
COPD/Asthma	33 (12.7%)
Immune deficiency	15 (5.8%)
Chronic liver disease	1 (0.4%)
Chronic kidney disease	4 (1.5%)
Diabetes	68 (26.2%)
Neuromuscular disease	3 (1.2%)

Note

Data are median (IQR) or n (%). COPD: chronic obstructive pulmonary disease; ICU: intensive care unit; IQR: interquartile range.

^a^ Data available in 230 patients.

A total of 77 (29.6%) patients received chloroquine phosphate before or during ICU admission. Such treatment was used off‐label since early reports suggested an association of chloroquine phosphate use with enhanced viral clearance and attenuated disease progression.^8^ Treatment with chloroquine phosphate was discontinued on 2 April 2020 by recommendation from The Swedish Medical Product Agency. The use of other antiviral or immunomodulation drugs was uncommon. Among the 260 patients, 225 (86.5%) received invasive mechanical ventilation for a median duration of 14 (IQR 9‐20) days, and 59 (22.7%) received renal replacement therapy in the ICU. Median ICU length of stay for all patients until study end point was 14 days (IQR 8‐21) (Table 2).

**Table 2:** Specific treatment and ICU support until study end point

Variable	All patients (n = 260)
Specific treatment^a^	
Chloroquine phosphate	77 (29.6%)
Hydroxychloroquine	1 (0.4%)
Tocilizumab	22 (8.5%)
Anakinra	1 (0.4%)
Lopinavir/ritonavir	1 (0.4%)
Remdesivir	1 (0.4%)
Highest level of respiratory support in the ICU	
Noninvasive ventilation or High‐flow oxygen	35 (13.5%)
Invasive ventilation	225 (86.5%)
Duration of invasive mechanical ventilation, days	14 (9‐20)
Renal replacement therapy	59 (22.7%)
ICU length of stay, days^b^	14 (8‐21)

Note

Data are median (IQR) or n (%). ICU: intensive care unit; IQR: interquartile range.

^a^ Specific treatment administered before and/or during ICU admission.

^b^ Including ICU length of stay preceding admission to the Karolinska University Hospital ICUs.

At the study end point (30 April 2020), 60 (23.1%) patients had died, 62 (23.9%) were discharged home, 47 (18.1%) were discharged to hospital wards, 29 (11.2%) were discharged to another health care facility (ward in another hospital, rehabilitation facility or nursing home) and 62 (23.9%) were still treated in ICU.

Mortality and ICU support for patients with data on definite outcomes (discharged alive from ICU or died; n = 198) and for patients still in ICU (n = 62) are presented in Table 3. Among the 198 patients with data on definite outcomes, ICU length of stay was 12 (IQR 6‐18) days, 163 (82.3%) received mechanical ventilation, 28 (14.1%) received renal replacement therapy, 60 (30.3%) died and 62 (31.3%) were discharged home. Mortality for patients younger than 40 years was 15.7% (3/19). The corresponding mortality for patients ≥ 70 years was 64.3% (18/28). Time to death within 120 days is displayed in Figure S1 in Electronic Supplementary Material 1.

**Table 3:** Outcomes and ICU support for patients with definite outcome (discharged alive from ICU or dead), or still in ICU at study end point, by age interval

	Patients discharged alive from ICU or dead at study end point						Patients still in ICU at study end point			
	Died		ICU length of stay, days	Renal replacement therapy	Mechanical ventilation	Mechanical ventilation duration, days	No.	ICU length of stay, days	Renal replacement therapy	Mechanical ventilation duration, days^a^
	Male	Female								
Age interval, years										
<40	2/14 (14.3%)	1/5 (20.0%)	9 (6‐14)	1/19 (5.3%)	13/19 (68.4%)	12 (8‐17)	5	25 (12‐25)	3/5 (60.0%)	22 (12‐25)
40‐49	4/22 (18.2%)	1/4 (25.0%)	13 (7‐20)	3/26 (11.5%)	23/26 (88.5%)	12 (9‐19)	5	28 (20‐28)	3/5 (60.0%)	28 (20‐28)
50‐59	13/50 (26.0%)	2/12 (16.7%)	12 (6‐18)	11/62 (17.7%)	49/62 (79.0%)	11 (7‐18)	23	23 (17‐28)	10/23 (43.5%)	22 (16‐28)
60‐69	15/49 (30.6%)	4/14 (28.6%)	13 (7‐18)	11/63 (17.5%)	55/63 (87.3%)	12 (8‐16)	25	24 (18‐27)	12/25 (48.0%)	24 (17‐27)
≥70	16/23 (69.6%)	2/5 (40.0%)	10 (5‐15)	2/28 (7.1%)	23/28 (82.1%)	11 (5‐15)	4	21 (20‐31)	3/4 (75.0%)	20 (19‐31)
All	50/158 (31.7%)	10/40 (25.0%)	12 (6‐18)	28/198 (14.1%)	163/198 (82.3%)	12 (8‐17)	62	24 (18‐28)	31/62 (50.0%)	22 (16‐27)

Note

Data are median (IQR) or n (%). ICU: intensive care unit; IQR: interquartile range.

^a^ All 62 patients who remained in ICU at study endpoint received invasive mechanical ventilation.

Among the 62 patients who were still in ICU at study end point, we observed a median ICU length of stay and duration of mechanical ventilation above 20 days and use of renal replacement in more than 40% across all age groups.

**Figure 2:**
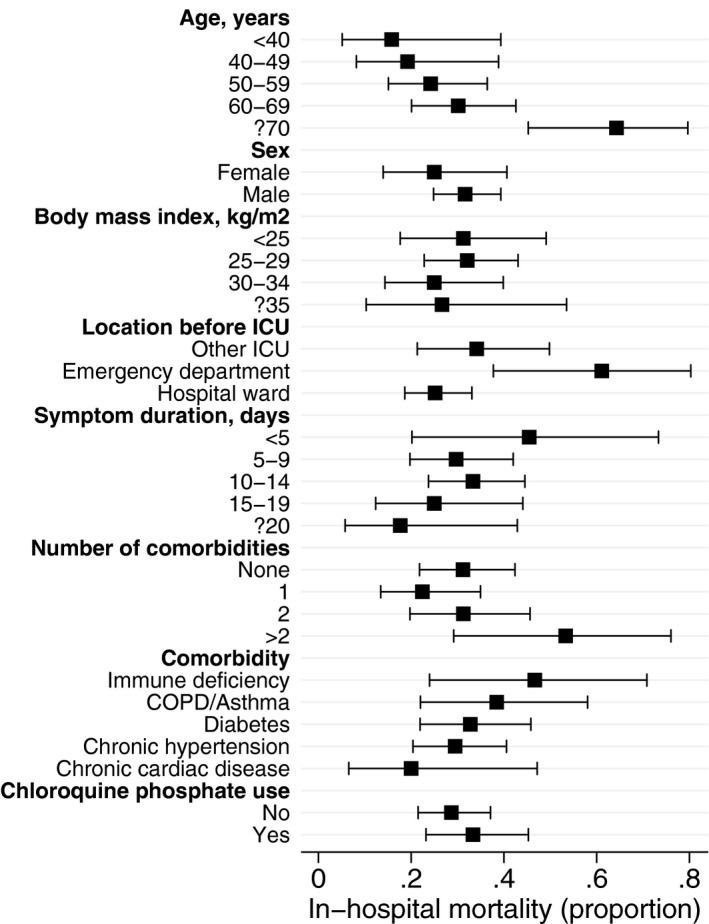
Proportion (95% CI) in‐hospital mortality for patients with definite outcome (discharged alive from ICU or dead) across subgroups. COPD, chronic obstructive pulmonary disease

The proportion (95% CI) of patients who died in hospital by age interval, sex, BMI interval, location before ICU admission, symptom duration, number and type of comorbidity and chloroquine phosphate use are summarized in Figure 2. Additional outcomes by subgroup are presented in the Supplementary Appendix (Table S1 to Table S5 in Electronic Supplementary Material 1).

On multivariable logistic regression analysis, older age (OR 1.07 per year, 95% CI 1.03‐1.10, *P* < .001) and admission from the emergency department (OR 4.57, 95% CI 1.55‐13.47, *P* = .006) were significantly associated with higher mortality. The adjusted OR for log symptom duration was 0.63 (95% CI 0.36‐1.09, *P* = .10). We observed no independent association of sex, number of comorbidities or chloroquine phosphate use with mortality (Table 4). Similar risk estimates were obtained when assessing 120‐day mortality in the multivariable Cox regression analysis (Table S6 in Electronic Supplementary Material 1).

**Table 4:** Univariate and multivariable logistic regression analysis showing the association with mortality

Variable	Univariate analysis		Multivariable analysis^a^	
	Odds ratio (95% CI)	*P* Value	Odds ratio (95% CI)	*P* Value
Age, years	1.06 (1.03‐1.09)	<.001	1.07 (1.03‐1.10)	<.001
Sex				
Female	1.00		1.00	
Male	1.39 (0.63‐3.06)	.42	1.34 (0.57‐3.15)	.51
Log symptom duration, days	0.72 (0.44‐1.17)	.19	0.63 (0.36‐1.09)	.10
Number of comorbidities				
None	1.00		1.00	
One or more	0.94 (0.50‐1.74)	.83	0.64 (0.31‐1.31)	.22
Location before ICU admission				
Ward or other ICU	1.00		1.00	
Emergency department	4.20 (1.54‐11.45)	.005	4.57 (1.55‐13.47)	.006
Chloroquine phosphate use				
No	1.00		1.00	
Yes	1.24 (0.66‐2.33)	.50	0.96 (0.47‐1.93)	.90

^a^ Area under the curve 0.73.

## 4 DISCUSSION

We studied the first 260 patients with COVID‐19 admitted to 9 ICUs in a tertiary hospital in Stockholm, Sweden. In this cohort the majority was men, median age was 59 years and 60% had at least one comorbidity. Among 198 patients with data on definite outcomes (discharged alive from ICU or death), mortality was 30.3%, median ICU length of stay was 14 days and 31.3% were discharged home. On adjusted analysis, we found a significant association of older age and admission from the emergency department with higher mortality.

While most European countries share the same goal of ensuring and controlling health care and limit economic consequences of the COVID‐19 pandemic, Sweden has taken a slightly different route to reach these common goals. In contrast to political and governmental reinforcement of country‐ or region‐wide lockdowns, Sweden have built its strategy on a combination of binding regulations and non‐political nation‐wide voluntary restrictions and quarantine recommendations including social distancing, as decided by national health authorities with a focus on protecting the elderly and other risk groups.^9^ Currently (as of 30 April 2020), Sweden has an estimated viral spread and mortality comparable with other European countries, yet higher than other Scandinavian countries such as Norway, Denmark and Finland. Sweden have reached a plateau on the viral spread curve, with an estimated R number of 1 at present.^10^

We observed a lower mortality compared to ICU cohorts from China, Italy and the US.^2^, ^3^, ^4^, ^11^ It seems that illness severity from SARS‐CoV‐2 varies substantially in different patient groups, with the highest mortality reported in older men with multiple coexisting diseases.^11^, ^12^ Mortality differences in ICU‐treated patients with COVID‐19 may have several potential explanations. First, ICU admission threshold have a great impact on survival. ICU capacity was not exceeded at Karolinska at any time. Our patients were slightly younger than reported in other ICU cohorts.^2^, ^4^, ^13^ However, older (70‐80 years) men had similar mortality as observed in Italy.^12^ Second, 53 (20.4%) of our patients were admitted from other ICUs. Critically ill patients eligible for transportation between hospitals are likely in a relatively stable clinical condition. However, mortality was slightly higher in patients admitted from other ICUs than in patients admitted from hospital wards suggesting that this cohort did not improve overall mortality. Third, we noted a relationship between symptom duration preceding ICU admission and mortality. Although this relationship did not reach statistical significance, short symptom duration likely represents a more aggressive disease course. Finally, we observed that patients receiving chloroquine phosphate had longer ICU stay, were twice as likely to receive dialysis, and had slightly higher mortality than patients not receiving this treatment. This finding should be interpreted with caution, as treatment was not given in a randomized fashion and no association between chloroquine phosphate use and mortality was observed in the multivariable analysis.

This study has limitations. First, the single‐hospital design limits generalizability. However, as of 20 April 2020 approximately one quarter of all patients with COVID‐19 admitted to Swedish ICUs had been treated at the Karolinska University Hospital. Second, at study end point, one quarter of the patients were still treated in ICU. These patients have an extended length of stay, a high proportion of renal replacement therapy, and potentially different outcomes than those included in our outcome analysis. Finally, information on severity of comorbidity and socioeconomic factors, which may impact outcome in critically ill patients, were not available in our data set. However, the study is based on validated data with available clinical outcome data for all patients.

## 5 CONCLUSIONS

In this study of critically ill patients with COVID‐19 admitted ICUs in a tertiary hospital in Stockholm, Sweden, presenting baseline characteristics and clinical course, mortality was 30.3%. Older age and admission from the emergency department were associated with higher mortality.

## 6 THE KAROLINSKA INTENSIVE CARE COVID‐19 STUDY GROUP

Christina Agvald Öhman, MD, PhD; Christian Ahlstedt, MD; Erzsébet Bartha, MD, PhD; Indir Becic, BIA; Max Bell, MD, PhD; Håkan Björne, MD, PhD; Jonas Blixt, MD; Olof Brattström, MD, PhD; Francesca Campoccia‐Jalde, MD, PhD; Janelle Cederlund, RN; Eva Christensson, MD; Johan Creutzfeldt, MD, PhD; Oili Dahl, CRNC, PhD; Bijan Darvish, MD, PhD; Lars I. Eriksson, MD, PhD; Mikael Eriksson, MD, PhD; Petter Eriksson, MD; Ola Friman, CRNC; Thomas Fux, MD, PhD; Janis Gotsis, MD; Anna Granström, CRNA; Jonatan Grip, MD, PhD; Anil Gupta, MD, PhD; Viveca Hambäck Hellkvist, CRNC; Elisabeth Hellgren, CRNC; Manne Holm, MD, PhD; Caroline Hällsjö Sander, MD, PhD; Cathrin Hällström, MD; Anna Januszkiewicz, MD; Malin Jonsson Fagerlund, MD, PhD; Kristina Kilsand, CRNC; David Konrad, MD, PhD; Ulrika Ljung Faxen, MD, PhD; Emma Larsson, MD, PhD; Andreas Liljekvist, MD; Lisbet Meurling, MD, PhD; Johan Mårtensson, MD, PhD; David Nelson, MD, PhD; Björn Nilsson, MD; Ulrica Nilsson, CRNA, PhD; Åke Norberg, MD, PhD; Anders Oldner, MD, PhD; Björn Persson, MD, PhD; Johan Petersson, MD, PhD; Claire Rimes Stigare, MD, PhD; Olav Rooyackers, PhD; Peter Rudberg, MD, PhD; Susanne Rysz, MD; Anna Schening, RN; Anna Somell, MD, PhD; Martin Sundström Rehal, MD; Nicolas Tardif, PhD; Inga Tjäder, MD, PhD; Jan van der Linden, MD, PhD; Eddie Weitzberg, MD, PhD.

## CONFLICT OF INTEREST

The authors report no conflicts of interest.

## Supporting information

Supplementary Material
