# Supplementary material for: Characteristics and outcomes of patients with COVID‐19 admitted to ICU in a tertiary hospital in Stockholm, Sweden
Source: Acta Anaesthesiol Scand. 2020 Sep 15;65(1):76–81. doi: 10.1111/aas.13694 (PMC7756749; doi:10.1111/aas.13694)
Supplement: Supplementary file 1 — Supplementary Material [file AAS-65-76-s001.docx]

**Electronic Supplementary Material 1**

Characteristics and outcomes of patients with COVID-19 admitted to ICU in a tertiary hospital in Stockholm, Sweden. Emma Larsson, MD, PhD; Olof Brattström, MD, PhD; Christina Agvald-Öhman, MD, PhD; Jonathan Grip, MD, PhD; Francesca Campoccia Jalde, MD, PhD; Kristoffer Strålin, MD, PhD; Pontus Nauclér, MD, PhD; Anders Oldner, MD, PhD; David Konrad, MD, PhD; Björn P. Persson, MD, PhD; Lars I. Eriksson, MD, PhD; Johan Mårtensson, MD, PhD

Content

[Table S1. Outcomes and ICU support for patients discharged alive from ICU or dead at study end point, by comorbidities. 2](#_Toc48647625)

[Table S2. Outcomes and ICU support for patients discharged alive from ICU or dead at study end point, by body mass index. 3](#_Toc48647626)

[Table S3. Outcomes and ICU support for patients discharged alive from ICU or dead at study end point, by duration of symptoms before ICU admission. 4](#_Toc48647627)

[Table S4. Outcomes and ICU support for patients discharged alive from ICU or dead at study end point, by location before ICU admission. 5](#_Toc48647628)

[Table S5. Outcomes and ICU support for patients discharged alive from ICU or dead at study end point, by chloroquine phosphate use. 6](#_Toc48647629)

[Table S6. Univariate and multivariable Cox regression analysis showing the association with 120-day mortality among all 260 patients 6](#_Toc48647630)

[Figure S1. Probability of survival during 120 days since ICU admission among the 260 patients. 7](#_Toc48647631)

# Table S1. Outcomes and ICU support for patients discharged alive from ICU or dead at study end point, by comorbidities.

|  | **Patients discharged alive from ICU or dead at study end point** | | | |
| --- | --- | --- | --- | --- |
|  | **Died, No./No. (%)** | **ICU length of stay, median (IQR), d** | **Dialysis,**  **No./No. (%)** | **Mechanical ventilation duration, median (IQR), d** |
| **No comorbidity** | 24/77 (31.2) | 11 (6-18) | 12/77 (15.6) | 12 (8-17) |
| **1 comorbidity** | 13/58 (22.4) | 12 (7-17) | 8/58 (13.8) | 12 (8-16) |
| **2 comorbidities** | 15/48 (31.3) | 10 (5-16) | 6/48 (12.5) | 11 (9-15) |
| **>2 comorbidities** | 8/15 (53.3) | 11 (5-22) | 2/15 (13.3) | 13 (6-22) |
| **Hypertension** | 23/78 (29.5) | 11 (6-17) | 11/78 (14.1) | 11 (8-16) |
| **Cardiac disease** | 3/15 (20.0) | 11 (4-14) | 1/15 (6.7) | 12 (9-17) |
| **COPD/Asthma** | 10/26 (38.5) | 9 (6-17) | 1/26 (3.9) | 11 (6-16) |
| **Immune disease** | 7/15 (46.7) | 17 (6-24) | 1/15 (6.7) | 13 (6-21) |
| **Liver disease** | 1/1 (100) | 11 (11-11) | 1/1 (100) | 11 (11-11) |
| **Kidney disease** | 4/4 (100) | 7 (5-16) | 2/4 (50.0) | 8 (5-23) |
| **Diabetes** | 19/58 (32.8) | 13 (5-18) | 8/58 (13.8) | 12 (9-18) |
| **Neuromuscular disease** | 1/3 (33.3) | 16 (13-32) | 2/3 (66.7) | 14 (11-33) |
| Abbreviations: COPD, chronic obstructive pulmonary disease; ICU, intensive care unit; IQR, interquartile range. | | | | |

# Table S2. Outcomes and ICU support for patients discharged alive from ICU or dead at study end point, by body mass index.

|  | **Patients discharged alive from ICU or dead at study end point** | | | |
| --- | --- | --- | --- | --- |
|  | **Died, No./No. (%)** | **ICU length of stay, median (IQR), d** | **Dialysis,**  **No./No. (%)** | **Mechanical ventilation duration, median (IQR), d** |
| **BMI interval, kg/m^2^** |  |  |  |  |
| **<25** | 10/32 (31.3) | 14 (7-18) | 5/32 (15.6) | 12 (8-18) |
| **25-29** | 26/81 (32.1) | 12 (6-17) | 13/81 (16.1) | 12 (9-17) |
| **30-34** | 11/44 (25.0) | 12 (7-19) | 7/44 (15.9) | 12 (9-18) |
| **≥35** | 4/15 (26.7) | 10 (7-16) | 1/15 (6.7) | 12 (6-15) |
| Abbreviations: BMI, body mass index; ICU, intensive care unit; IQR, interquartile range. | | | | |

# Table S3. Outcomes and ICU support for patients discharged alive from ICU or dead at study end point, by duration of symptoms before ICU admission.

|  | **Patients discharged alive from ICU or dead at study end point** | | | |
| --- | --- | --- | --- | --- |
|  | **Died, No./No. (%)** | **ICU length of stay, median (IQR), d** | **Dialysis,**  **No./No. (%)** | **Mechanical ventilation duration, median (IQR), d** |
| **Symptom duration interval, d** |  |  |  |  |
| **<5** | 5/11 (45.5) | 10 (2-12) | 1/11 (9.1) | 10 (8-12) |
| **5-9** | 19/64 (29.7) | 12 (7-18) | 12/64 (18.8) | 12 (7-18) |
| **10-14** | 26/78 (33.3) | 11 (5-16) | 11/78 (14.1) | 12 (8-17) |
| **15-19** | 7/28 (25.0) | 11 (7-18) | 4/28 (14.3) | 9 (7-16) |
| **≥20** | 3/17 (17.7) | 15 (10-20) | 0/17 (0) | 14 (9-20) |
| Abbreviations: ICU, intensive care unit; IQR, interquartile range. | | | | |

# Table S4. Outcomes and ICU support for patients discharged alive from ICU or dead at study end point, by location before ICU admission.

|  | **Patients discharged alive from ICU or dead at study end point** | | | |
| --- | --- | --- | --- | --- |
|  | **Died, No./No. (%)** | **ICU length of stay, median (IQR), d** | **Dialysis,**  **No./No. (%)** | **Mechanical ventilation duration, median (IQR), d** |
| **Location before ICU admission** |  |  |  |  |
| **Other ICU** | 14/41 (34.2) | 17 (11-21) | 7/41 (17.1) | 13 (9-19) |
| **Emergency department** | 11/18 (61.1) | 9 (6-16) | 6/18 (33.3) | 14 (6-18) |
| **In hospital admission** | 35/139 (25.2) | 10 (5-16) | 15/139 (10.8) | 11 (7-155 |
| Abbreviations: ICU, intensive care unit; IQR, interquartile range. | | | | |

# Table S5. Outcomes and ICU support for patients discharged alive from ICU or dead at study end point, by chloroquine phosphate use.

|  | **Patients discharged alive from ICU or dead at study end point** | | | |
| --- | --- | --- | --- | --- |
|  | **Died, No./No. (%)** | **ICU length of stay, median (IQR), d** | **Dialysis,**  **No./No. (%)** | **Mechanical ventilation duration, median (IQR), d** |
| **Received chloroquine phosphate** |  |  |  |  |
| **Yes** | 23/69 (33.3) | 14 (8-21) | 15/69 (21.7) | 13 (9-19) |
| **No** | 37/129 (28.7) | 10 (6-16) | 13/129 (10.1) | 11 (7-16) |
| Abbreviations: ICU, intensive care unit; IQR, interquartile range. | | | | |

# Table S6. Univariate and multivariable Cox regression analysis showing the association with 120-day mortality among all 260 patients

|  | **Univariate analysis** | | **Multivariable analysis^a^** | |
| --- | --- | --- | --- | --- |
| **Variable** | **Hazard ratio**  **(95% CI)** | **P Value** | **Hazard ratio**  **(95% CI)** | **P Value** |
| **Age, years** | 1.06 (1.03-1.08) | <0.001 | 1.06 (1.04-1.09) | <0.001 |
| **Sex** |  |  |  |  |
| **Female** | 1.00 |  | 1.00 |  |
| **Male** | 1.55 (0.82-2.93) |  | 1.55 (0.81-2.97) | 0.18 |
| **Log symptom duration, days** | 0.93 (0.65-1.32) |  | 0.81 (0.56-1.19) | 0.28 |
| **Number of comorbidities** |  |  |  |  |
| **None** | 1.00 |  | 1.00 |  |
| **One or more** | 0.84 (0.54-1.32) |  | 0.68 (0.43-1.08) | 0.11 |
| **Location before ICU admission** |  |  |  |  |
| **Ward or other ICU** | 1.00 |  | 1.00 |  |
| **Emergency department** | 2.09 (1.17-3.74) |  | 2.12 (1.18-3.81) | 0.01 |
| **Chloroquine phosphate use** |  |  |  |  |
| **No** | 1.00 |  | 1.00 |  |
| **Yes** | 1.19 (0.74-1.92) |  | 0.98 (0.60-1.59) | 0.92 |
| ^a^Test of proportional-hazards assumption, P=0.31 | | | | |

# Figure S1. Probability of survival during 120 days since ICU admission among the 260 patients.
